# Study on the Mechanical Properties and Binding Behavior of Chloride in Cement Paste Under Premixed High Concentration of Chloride Ions

**DOI:** 10.3390/ma18194465

**Published:** 2025-09-25

**Authors:** Aiqin Wang, Xixian Du, Gang Li, Aoli Cao, Yuwei Ma, Yang Zhou

**Affiliations:** 1 College of Water Conservancy & Architectural Engineering, Shihezi University, Shihezi 832000, China; wangaiqin@shzu.edu.cn (A.W.); xxdu@stu.shzu.edu.cn (X.D.); aolicao@stu.shzu.edu.cn (A.C.); myw819@shzu.edu.cn (Y.M.); zhougroup@shzu.edu.cn (Y.Z.); 2 Key Laboratory of Cold and Arid Regions Eco-Hydraulic Engineering of Xinjiang Production & Construction Corps, Shihezi 832000, China

**Keywords:** binding capacity, cement paste, Friedel’s salt, bound Cl^−^, compressive strength

## Abstract

Chloride erodes steel bars through concrete pores, seriously affecting the durability of reinforced concrete structures. Improving the binding ability to chloride is an important measure. We explored the effects of W/C, curing age, and premixed Cl^−^ concentration on the compressive strength and Cl^−^ binding capacity in cement pastes. The results indicate that a premixed 5% concentration of Cl^−^ can improve the compressive strength, whereas an excessive Cl^−^ negatively impacts the mechanical properties. The total Cl^−^ content in cement pastes is a crucial factor that influences the binding ability of Cl^−^. When the total Cl^−^ content is within 2% (i.e., the premixed Cl^−^ concentration is 5%), the cement paste has a strong binding ability of Cl^−^. W/C and curing age indirectly affect the binding ability by affecting the total Cl^−^ content. Furthermore, with the increase in content of Cl^−^, the adsorption content of Cl^−^ by C-S-H increased, while the proportion of Cl^−^ bound by Fs to the total bound Cl^−^ initially declines and then tends to stabilize. It is worth noting that a premixed concentration of 5% is a “safety limit” for cement paste, but for reinforced concrete, the presence of free Cl^−^ above normative thresholds should not be underestimated.

## 1. Introduction

The corrosion of steel bars in reinforced concrete structures can significantly impact the service life of a building, with chloride ions (Cl^−^) being one of the primary contributors to this corrosive phenomenon [1]. However, in coastal and salt lake areas, due to the lack of freshwater resources, seawater and salt lake water have good prospects for use in concrete buildings. However, this will significantly increase the content of Cl^−^ inside concrete, which will have a significant influence on the performance of reinforced concrete structures [2]. The Cl^−^ content is a key factor affecting the corrosion of steel bars. Once the Cl^−^ content on the surface of steel bars reaches the corrosion threshold, it will cause corrosion of the steel bars in concrete, leading to the concrete cracking and peeling, and to delamination between steel bars. According to British standards [3], a Cl^−^ content of less than 0.4% is safe for reinforced concrete structures, but the Cl^−^ content is not a fixed value, but instead a threshold range influenced by multiple factors. Ki Yong Ann reviewed the Cl^−^ threshold levels reported by different authors under different conditions [4]. The hydration products of cementitious materials (cement) or supplementary cementitious materials (such as burnt clay [5], etc.) in reinforced concrete can combine with Cl^−^ entering the interior of the concrete. The improvement of the binding ability of Cl^−^ in cementitious materials will hinder their migration to the surface of steel bars [6]. Consequently, in order to control and prevent early deterioration of reinforced concrete structures, studying the performance alterations of concrete under premixed Cl^−^ conditions is of great significance.

Cl^−^ ions in concrete are present as free Cl^−^ or bound Cl^−^, with the latter being largely innocuous to the durability of concrete structures [7]. Research indicates that the binding behavior is predominantly associated with cement composition, which primarily binds Cl^−^ through chemical binding and physical adsorption. Specifically, chemical binding involves binding Cl^−^ to monosulphoaluminate (AFm, 3CaO·Al_2_O_3_·CaSO_4_·12H_2_O), which forms Friedel’s salt (Fs, 3CaO·Al_2_O_3_·CaCl_2_·10H_2_O) [8]. Physical adsorption denotes the process whereby Cl^−^ is adsorbed onto the surface or between layers of cement hydration products, particularly C-S-H [9]. In ordinary Portland cement (OPC) systems, chemically bound Cl^−^ ions exhibit greater stability and reduced susceptibility to decomposition [10], whereas physically adsorbed Cl^−^ ions are reversible [11]. The binding behavior of chloride influences the hydration process of OPC when Cl^−^ ions are mixed into the concrete through premixing [12]. According to Kaushik [13] and Mbadike [14], freshwater concrete outperforms seawater concrete in terms of compressive strength. For example, at a W/C of 0.5, the average compressive strength of concrete prepared with freshwater ranged from 25.05 to 38.13 N/mm^2^, compared to a range of 23.58 to 36.03 N/mm^2^ for concrete mixed using saltwater. However, Shi [15] and Taylor [16] found that the compressive strength increased by 22% when mixed with seawater.

The binding efficacy of cement on Cl^−^ can markedly improve the durability of concrete structures. Therefore, researchers have undertaken numerous investigations to analyze the factors affecting the binding ability of Cl^−^ [17]. Some studies indicate that variations in Cl^−^ concentration can substantially impact the binding capacity [18]. Chang [19] concluded that fluctuations in Cl^−^ concentration influence the stability of Fs. Hirao [20] demonstrated an enhancement in Cl^−^ adsorption capacity of C-S-H with rising Cl^−^ concentration. However, there is a dispute over the specific scope to which chemically bounded Cl^−^ and physically adsorbed Cl^−^ are influenced by the concentration of Cl^−^ [19]. In addition, factors such as W/C, the age of curing, salt ion type, mineral admixtures and electric field have an indirect impact on the binding capacity of Cl^−^ [21]. Studies indicate that the presence of sulfate ions can produce expansion products, thereby affecting the diffusion of Cl^−^ [22]; mineral admixtures can promote the formation of C-S-H, thus enhancing the solidification ability [23]; and the electric field will reduce the curing effect [24]. However, Zhao et al. [25] discovered in their research that the binding ratio of Cl^−^ progressively rises as the W/C decreases. However, Zhang et al. [26] proposed that the cement-based materials’ ability to bind Cl^−^ increases as the W/C increases. Consequently, in light of these conflicting conclusions, further research is warranted to elucidate the factors governing the binding capacity of Cl^−^ ions and their influencing mechanisms.

At present, the existing literature mainly focuses on the study of Cl^−^ binding ability through penetration tests (i.e., the penetration of external Cl^−^ into concrete), and the Cl^−^ concentration is relatively low. However, research on the mechanical properties and Cl^−^ binding ability in case of premixed high concentration Cl^−^ is very limited (i.e., Cl^−^ are already evenly distributed in concrete). Therefore, in this study, considering the high Cl^−^ concentration in salt ponds and high salinity lakes, such as the Great Salt Lake in the United States with a salinity up to 340 g L^−1^ [27], in order to purely evaluate the binding ability of materials, and exclude the influence of bone materials and other factors, the variations in the binding capacity of Cl^−^ across different premixed Cl^−^ concentrations, W/C, and curing ages are studied by preparing a cement paste with a high concentration of Cl^−^ to elucidate the alteration rule in the binding behavior of Cl^−^. The impact of Cl^−^ binding on the mechanical properties of cement paste was analyzed through strength tests at a macroscopic level. Additionally, microanalysis was conducted to uncover the Cl^−^ binding mechanism. Simultaneously, XRD and TG tests were used to analyze the hydration products qualitatively and quantitatively, calculate the content of binding Cl^−^ from Fs and C-S-H, and examine how chemically bound versus physically adsorbed Cl^−^ ions are distributed and vary. The findings can offer a theoretical foundation for the application of seawater as well as saline lake water in concrete construction.

## 2. Method

### 2.1. Materials

The OPC (Purchased from Tianneng Cement Co., Ltd, Xinjiang, China) utilized possessed a strength classification of 42.5 and a Cl^−^ content of less than 0.1 percent in the cement (expressed as a mass fraction of the cement). Analytically, pure NaCl (Purchased from Anxingshun Chemical Co., Ltd, Urumqi, Xinjiang, China. purity greater than 99.5%) was added to deionized water for the purpose of premixing Cl^−^. The chemical composition and performance parameters of OPC are displayed in Table 1 and Table 2, respectively.

### 2.2. Preparation and Curing

Cement pastes were created by combining OPC with deionized water that had been premixed with Cl^−^. The W/C were 0.3, 0.4, and 0.5, respectively. The quantities of the materials are detailed in Table 3. The solubility of NaCl in water at room temperature (20 °C) is 35.9 g per 100 mL, corresponding to a concentration of approximately 26.4%, while the concentration of Cl^−^ in saline lake water can be up to 20% or more. Consequently, to examine the impact of a high concentration of Cl^−^ on the ability of Cl^−^ to bind, the premixed Cl^−^ concentrations were taken as 5%, 10%, 15%, and 20% (expressed as the concentration of mixing water). NaCl was introduced into the deionized water and agitated extensively until fully dissolved. Subsequently, it was combined with cement and churned for a duration of 2 min using an electric mixer operating (Purchased from Bowang Enterprise, Shihezi, China) at 1800 rpm. Then, the mixture was transferred into molds and vibrated for a period of 2 min. The specimens were removed from molds after 24 h. They were then subjected to curing for 28, 56, 90, and 120 days, respectively, under the usual curing conditions of a temperature of 20 ± 2 °C and a humidity level exceeding 95%.

### 2.3. Test Methods for Compressive Strength and Bound Content of Cl^−^

**Figure 1:**
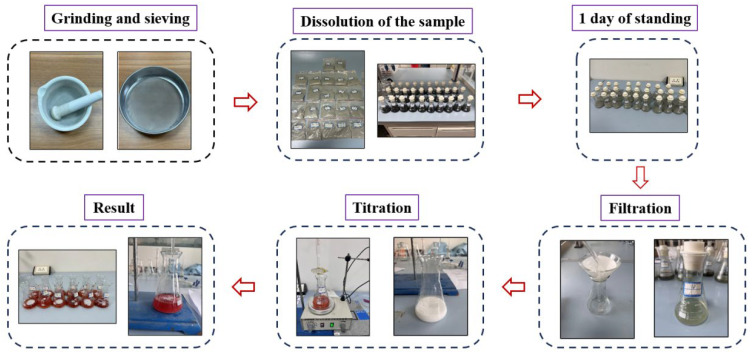
Chemical titration process.

The cubic compressive strength and the content of Cl^−^, both free and total, were examined in reference to the JTS/T 236-2019 specification [28]. A 2000 kN universal testing machine (Purchased from Testing Machine Group Co., Ltd, Jinan, China) was used for compressive strength testing. After curing the specimen to the specified age, it was taken out for testing in a timely manner. First, the surface of the specimen and the pressure plate of the universal testing machine were wiped. Then, the specimen was placed vertically on the pressure plate. The loading speed was 0.5 MPa/s. At specimen failure, the peak load was recorded. The specimen size used for compressive strength testing was 100 mm × 100 mm × 100 mm, and the strength value for the group was calculated as the average of the three test specimens. If either the maximum or minimum value deviated from the median by more than 15%, the median value was adopted as the compressive strength. If both the maximum and minimum values deviated from the median by more than 15%, the entire set of experimental results was discarded. Figure 1 shows the titration procedure. Initially, the samples underwent crushing, grinding, and sieving. Samples weighing 10 g were dissolved in deionized water and a 15% nitric acid solution. The Cl^−^ content that dissolved in acid (total Cl^−^ content) and in water (free Cl^−^ content) were determined by adding a 0.1 mol/L silver nitrate solution. The percentage of bound Cl^−^ was calculated using the formula [29]:

(1) $$C_{\mathrm{bound}}=C_{\mathrm{total}}-C_{\mathrm{free}}$$

where *C_bound_* is the bound Cl^−^ content, *C_total_* is the total Cl^−^ content, and *C_free_* is the free Cl^−^ content. *C_total_*, *C_bound_* and *C_free_* are all expressed as percentages of the mass of Cl^−^ in cement paste, i.e., the mass of Cl^−^ per gram of cement paste.

The binding capacity *R_b_* to Cl^−^ is the ratio of the bound Cl^−^ content to the total Cl^−^ content, as in Formula (2):

(2) $$R_{b}=\frac{C_{bound}}{C_{total}}$$

### 2.4. SEM, XRD and DTG Tests

Sample fragments with dimensions of 5–8 mm were characterized by SEM using a Su-8010 electron microscope. Before the test, the samples underwent gold-spraying treatment. An XRD analysis was carried out by a D/MAX 2000 X-ray diffractometer, with a scanning angle of 5° to 80° and a rate of 10°/min. The DTG analysis was performed by an SDT650 instrument, with an initial test temperature of 30 °C, a maximum temperature of 800 °C, and a temperature increase rate of 10 °C/min. The preparation procedure for powder samples utilized for the XRD analysis and the TG analysis is as follows: the collected particles and block samples are ground and sieved. Later, the sieved powder is placed in an oven to dry at 70 °C for a duration of 6 h to terminate hydration. Above tests are all conducted by the Compass Testing Center, Shanghai, China.

## 3. Results

### 3.1. Compressive Strength

**Figure 2:**
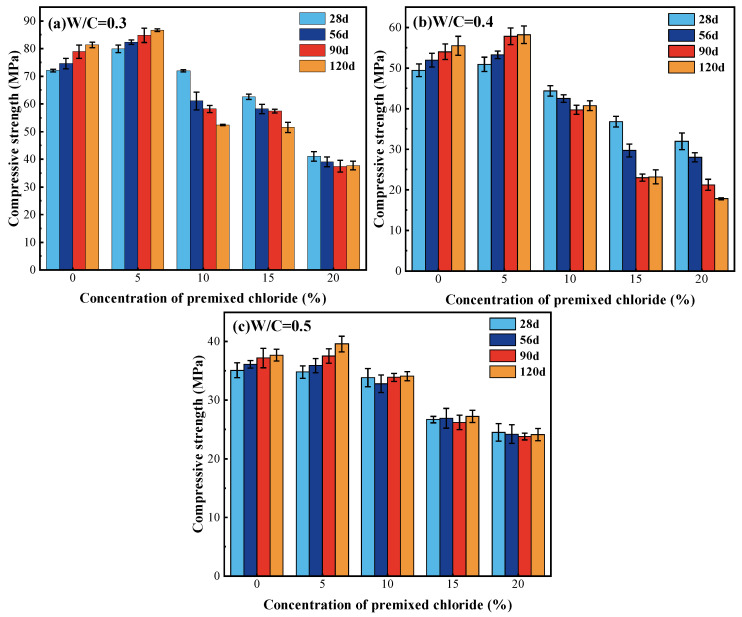
Effect of curing age and premixed Cl^−^ concentration on compressive strength. (**a**) W/C = 0.3, (**b**) W/C = 0.4, (**c**) W/C = 0.5.

Figure 2 illustrates variations in the compressive strength of cement paste due to the W/C, age of curing, and the concentration of premixed Cl^−^. The statistical analysis results show that the difference in compressive strength between groups is statistically significant (*p* < 0.05), indicating the reliability of the data. Overall, the compressive strength development at all curing ages was inversely correlated with the premixed Cl^−^ concentration, beyond a certain threshold. While concentrations up to 5% were beneficial, any further increase led to a decline, an effect that was markedly more evident in systems with a W/C of 0.3 or 0.4.

The W/C has a significant impact on compressive strength. Assuming the curing age and Cl^−^ concentration are held constant, when the W/C is at 0.3, the compressive strength is the highest, followed by 0.4, and 0.5 as the lowest.

The concentration of Cl^−^ is also one of the key factors influencing the strength. In the absence of premixed Cl^−^, compressive strength increases with curing age for different W/C values. At the W/C of 0.3, the strengths at 28 days and 120 days of curing are 72.1 MPa and 81.35 MPa, respectively. The compressive strength of cement paste after hydration for 28 days reaches around 90% of that after hydration for 120 days. The change in the low W/C (W/C = 0.3) system on strength is more pronounced when 5% Cl^−^ ions are added to the cement paste. The strength of the cement paste at 28 days and 56 days significantly increases, reaching a level similar to that of the strength at 90 days and 120 days without Cl^−^. This suggests that Cl^−^ can facilitate the hydration reaction of the cement and contribute to early strength improvement. The main mechanism involves the reaction of Cl^−^ with C_3_A in the cement clinker composition, forming the AFm phase (AFm-Cl^−^), which promotes the hydration reaction as follows [30]:

(3) $$C_{3}A+Ca^{2+}+2Cl^{-}+10H_{2}0\to 3CaO\cdot Al_{2}O_{3}\cdot CaCl_{2}\cdot 10H_{2}O$$

AFm-Cl^−^, also known as Fs, whose presence results from the reaction, fills the pores of the cement paste, leading to a more refined microstructure. This refined microstructure is the reason for the increased strength at 90 and 120 days. The compressive strength decreases as the W/C increases. This is due to a decrease in the amount of cementitious material in the system and an increase in porosity. Specifically, the strength at 28 days without premixing Cl^−^ decreases by 31.4% and 51.3% when the W/C is equal to 0.4 and 0.5, respectively, compared to W/C at 0.3. Furthermore, the reduction in system densities leads to a lessening impact of the premixed 5% concentration of Cl^−^ on the strength.

**Figure 3:**
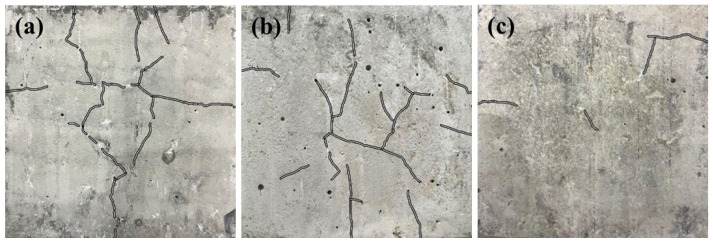
Apparent morphology of cementitious net paste with premixed Cl^−^ concentration of 20% and curing age of 120 d, (**a**) W/C = 0.3, (**b**) W/C = 0.4, (**c**) W/C = 0.5.

When the premixed Cl^−^ concentration reaches 10% or higher, the excessive presence of Cl^−^ has a negative impact on the compressive strength. When the concentration of Cl^−^ increases, compressive strength decreases continuously at all ages of curing. This decrease is primarily caused by the excess Cl^−^ accelerating the leaching of calcium from the hydration products. This leaching leads to an improved solubility of the hydration products in water and to an increase in porosity, which adversely affects the structure. On the other hand, the ongoing creation of Fs and the formation of crystalline NaCl due to the increasing concentration of premixed Cl^−^ have also resulted in structural damage. Decreasing the W/C leads to an increased density in the interior of the cement paste, resulting in more significant damage [17]. As a result, the decrease in strength is more pronounced at lower W/C (W/C = 0.3, 0.4). Figure 3a,b demonstrates that, with W/C at 0.3 and 0.4, the premixed Cl^−^ concentration is 20%, and the curing age is 120 days, the surface of the specimens exhibits multiple cracks. And the compressive strengths of the cement pastes decrease by 53.6% and 67.9%, respectively, compared to the strengths of the same age without premixed Cl^−^. This decrease in strength can be attributed to NaCl and Fs, which produce pore wall stress greater than the strength of the cement paste [31]. At a W/C of 0.5, the presence of NaCl and Fs has less impact on the strength of the specimen, resulting in fewer surface cracks. The compressive strength only drops by 36%, compared to the strengths of the same age without premixed Cl^−^, as depicted in Figure 3c.

Based on the analysis above, a Cl^−^ concentration of 5% is considered a safe limit, comparable to the concentration of Cl^−^ found in seawater [2]. Numerous studies have also indicated that the addition of seawater does not lead to significant structural defects in concrete but instead enhances its physical and mechanical properties [32]. The contradiction of existing research mainly focuses on the influence of Cl^−^ on concrete strength under different curing ages. Xiao et al. [33] concluded that Cl^−^ ions enhance strength at an early age, and Wang et al. [2] found that the compressive strength of seawater slurry increased by over 50% at 28 days. However, other studies have shown that Cl^−^ ions actually enhance strength at a later age but may slightly reduce strength at 28 days [34]. In this study, it was revealed that Cl^−^ ions exhibit varying effects on the strength of cement paste according to the W/C conditions. When the W/C is 0.3, Cl^−^ improved compressive strength at both early (28 days, 56 days) and late ages (90 days, 120 days). However, W/C values of 0.4 and 0.5, Cl^−^ mostly affected the improvement of late strength (90 days, 120 days).

### 3.2. SEM Analysis

**Figure 4:**
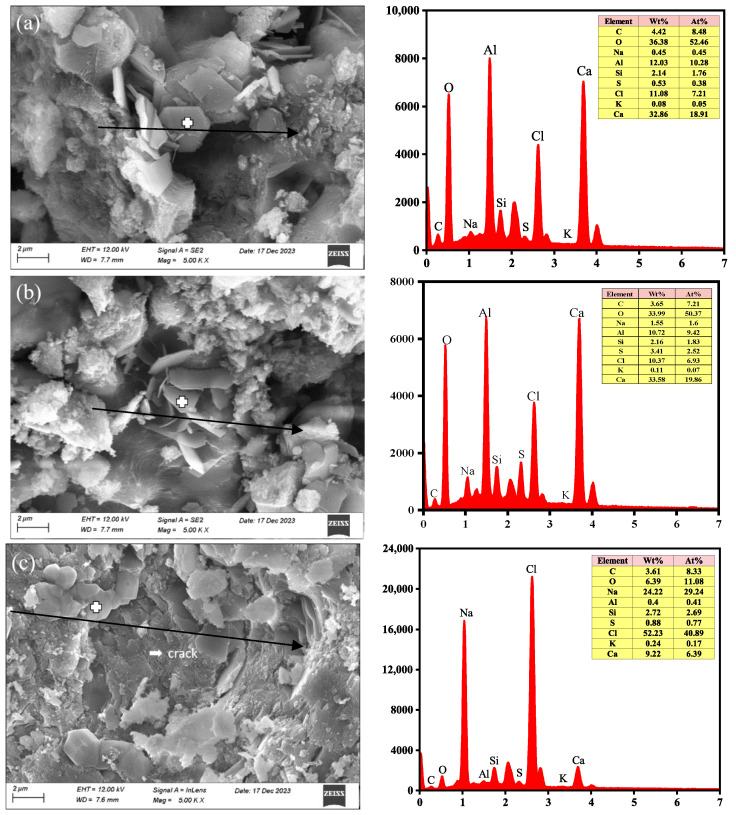
SEM-EDS results for cement paste, (**a**,**b**) 0.4-120d-5%, (**c**) 0.4-120d-20% (W/C—curing age—premixed Cl^−^ concentration).

Figure 4 displays the microscopic morphology of the inside of the cement paste when the W/C is 0.4, and the curing age is 120 days. The microscopic morphology of Fs under the electron microscope, as shown in Figure 4a,b, reveals a lamellar hexagonal morphology measuring approximately 2 μm. This observation aligns with existing studies [35]. Additionally, the EDS spectroscopy analysis identified the presence of oxygen (O), aluminum (Al), chlorine (Cl), and calcium (Ca) as the main elements in the sample. This further supports the conclusion that the observed lamellar hexagon is indeed Fs. Fs primarily exists in the cement paste in two forms: either within the pores or embedded in C-S-H. This presence enhances the overall compactness of the paste, which is also the reason why the compressive strength increases when a 5% concentration of Cl^−^ is premixed. Figure 4c shows that when the concentration of premixed Cl^−^ is 20%, NaCl crystals precipitate within the cement paste. These crystals are distinctly angular and bond together. An EDS spectroscopy analysis confirmed that the bonded substances in Figure 4c are NaCl crystals. The precipitation of NaCl crystals within the pores produces crystallization pressure, leading to severe harm to the pore structure. This process also leads to numerous microcracks, ultimately impacting the strength of the material. Therefore, when excessive NaCl is premixed, due to the reason that the cement pastes with low W/C are more compact internally, the NaCl in smaller pores is more easily saturated. Meanwhile, the formation of Fs and the precipitation of NaCl crystals can cause higher pressure on the pore walls, and the damage to the structural integrity of cement pastes becomes more severe.

### 3.3. XRD Analysis

**Figure 5:**
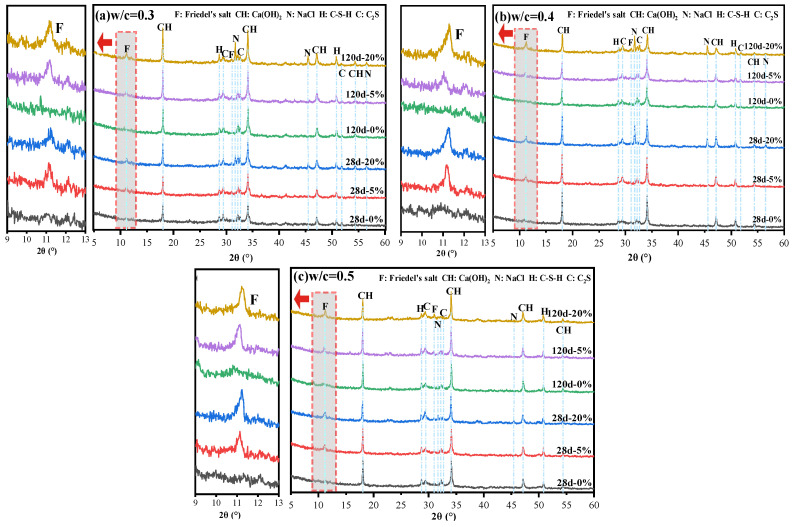
XRD patterns of cement paste at (**a**) W/C = 0.3, (**b**) W/C = 0.4, (**c**) W/C = 0.5. The main crystal phases are Friedel’s salt (F, PDF # 00-042-0488), calcium hydroxide (CH, PDF # 00-004-0733), sodium chloride (N, PDF # 00-005-0628), and dicalcium silicate (C, PDF # 00-033-0302); the peak located at about 29° (2θ) belongs to the C-S-H gel.

An XRD analysis of the hydration products in cement pastes, as shown in Figure 5, shows the characteristic peaks of Fs at approximately 2θ = 11.2° in the sample that is premixed with Cl^−^ [19]. This finding confirms that OPC binds Cl^−^ by forming Fs. Additionally, the variation in the type of hydration product indicated that the formation of Fs is reason for the increase in the compressive strength when premixed with 5% Cl^−^. When a 20% concentration of Cl^−^ is premixed to the cement paste, the XRD pattern shows characteristic peaks of NaCl. These peaks are more pronounced when the W/C is 0.3 or 0.4. However, while the W/C increases to 0.5, the peaks of NaCl weaken or disappear, mainly due to the increase in the W/C leading to higher porosity and an increase in the amount of Cl^−^ in the pore space [17]. These Cl^−^ ions primarily exist in the pore space in a free form rather than as NaCl precipitation. Therefore, the characteristic peaks of NaCl in the test samples are lower at a higher W/C (W/C = 0.5), a phenomenon consistent with the results obtained from the observations of the cement paste’s apparent morphology presented in Figure 3.

### 3.4. Distribution of Free and Bound Cl^−^

**Figure 6:**
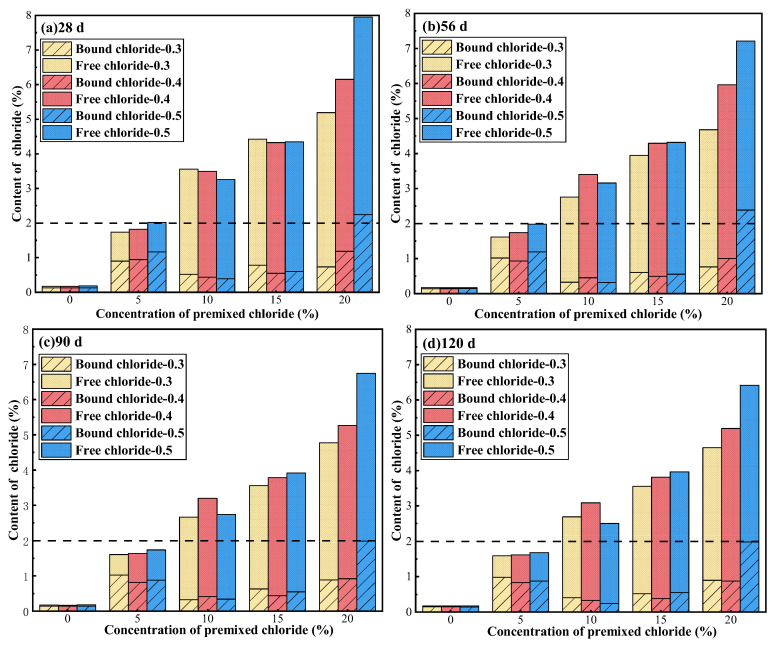
Content of bound and free Cl^−^ in cement paste at curing ages of (**a**) 28 d, (**b**) 56 d, (**c**) 90 d and (**d**) 120 d.

The distribution of free and bound Cl^−^ was acquired by chemical titration, as depicted in Figure 6. In the absence of premixed Cl^−^, a small quantity of Cl^−^ can be observed in the cement paste, primarily originating from the cement. When the Cl^−^ concentration is low, the effect of binding Cl^−^ is prominent, with a significantly higher content of bound Cl^−^ compared to free Cl^−^.

The total Cl^−^ content in the cement paste is around 2% when a 5% concentration of Cl^−^ is premixed, taking a substantial increase in both bound and free Cl^−^ content compared to a situation without premixing Cl^−^. Yet, the majority of Cl^−^ ions remain in the bound form, and the primary method of chemical binding for Cl^−^ is the formation of Fs, as revealed by the results of SEM and XRD analysis. As the curing age increases, the content of total Cl^−^ decreases slightly. This suggests that when the premixed Cl^−^ concentration is 5%, there is also the occurrence of NaCl crystal precipitation inside the cement paste with the hydration reaction of cement. However, the amount of precipitation is minimal and does not cause any structural damage [31]. On the contrary, the presence of NaCl crystals and Fs strengthens the compactness of the specimens and expands the compressive strength by filling of pores. At the curing ages of 28 days and 56 days, the highest levels of total Cl^−^ are observed when the W/C is 0.5, in which case the bound Cl^−^ content is also highest. However, as the curing age increases, the content of total Cl^−^ remains similar under all W/C conditions, maintaining around 1.6%, and when the W/C is 0.3, the content of bound Cl^−^ is highest (around 1%). This indicates that the total Cl^−^ content is a crucial factor impacting the binding capacity of Cl^−^. When the content of total Cl^−^ in the cement paste is less than 2%, a higher content of total Cl^−^ leads to a more pronounced binding behavior. However, when the content of total Cl^−^ is similar, the cement paste system with W/C = 0.3 exhibits a stronger effect of chloride binding due to the presence of a greater amount of cementitious materials.

When a premixed Cl^−^ concentration is in the range of 5–10% (exceeds 5%), the content of total Cl^−^ surpasses 2%, and the content of free Cl^−^ increases significantly, while the content of bound Cl^−^ decreases. This suggests that higher Cl^−^ content negatively impacts the binding ability of Cl^−^, leading to a decreased content of bound Cl^−^. Nevertheless, when total Cl^−^ content surpasses 2% (a Cl^−^ concentration of 10–20%), the bound Cl^−^ content rises as the total Cl^−^ content increases further. This suggests that the binding capacity of Cl^−^ does not diminish continuously with the increase in the total Cl^−^ content. Simultaneously, the increased content of Cl^−^ results in more serious precipitation of NaCl crystals. As the curing age increases, there is an apparent decrease in the content of total Cl^−^ at premixed Cl^−^ concentrations of 10%, 15%, and 20%. This change causes a shift in the distribution of Cl^−^, affecting both bound and free Cl^−^. Moreover, if the concentration of premixed Cl^−^ exceeds 5%, the Cl^−^ ions start to impact the overall structure of the cement paste adversely. Consequently, the structural integrity of the cement paste may also be important in influencing its ability to bind Cl^−^.

When the concentration of premixed Cl^−^ is below 20%, the influence of the W/C on both the total Cl^−^ content and the bound Cl^−^ content in the cement paste is not readily apparent. This may be attributed to the varying crystallization sites of NaCl. When the W/C is higher, the cement paste exhibits greater porosity and smaller pore size, resulting in the formation of more salt crystals on the surface of the cement. This reduces the internal Cl^−^ content. Consequently, the content of total Cl^−^ inside the cement paste is similar throughout the various W/C. Nevertheless, when premixed with a 20% concentration of Cl^−^, the pressure caused by the crystallization of NaCl and Fs within the fissures of the cement paste fractures the matrix [36], providing space for further precipitation of NaCl inside. Simultaneously, due to the higher density of the cement paste at lower W/C, the occurrence of matrix cracking is more severe. These fissures serve as a pathway for the precipitation of NaCl into the external environment. Consequently, reducing the W/C leads to a drop in the total Cl^−^ content within the cement paste, and the bound Cl^−^ content also lowers as the total Cl^−^ content falls.

In conclusion, the content of total Cl^−^ in the cement paste significantly influences its ability to bind Cl^−^. When the concentration of Cl^−^ in the mixing water is 5%, the cement paste binds Cl^−^ effectively. However, the content of free Cl^−^ still exceeds the threshold value for reinforcing steel corrosion [37]. Therefore, additional measures are required to reduce the total Cl^−^ content and enhance the capacity of binding Cl^−^.

### 3.5. Binding Capacity of Cl^−^

**Figure 7:**
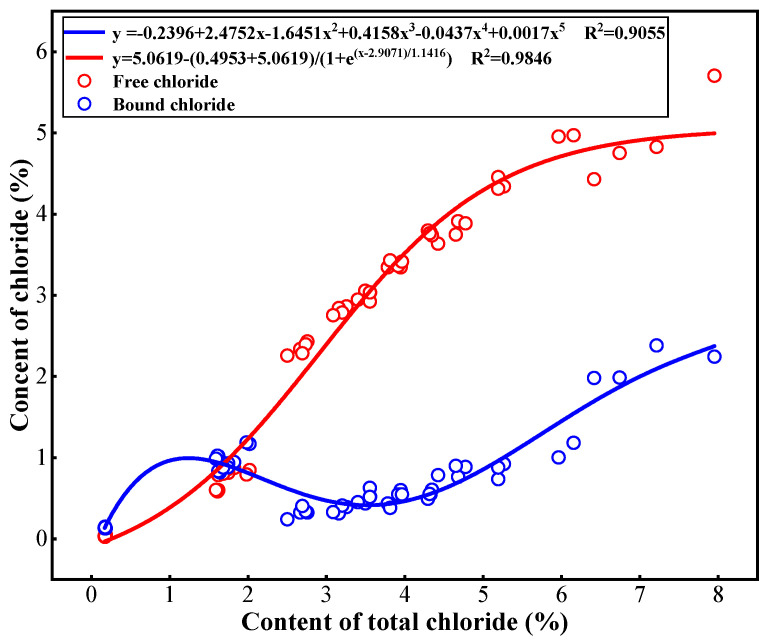
Relationship between total Cl^−^ and free, bound Cl^−^ in cement paste.

The relationship between total Cl^−^ and free/bound Cl^−^ in cement paste is fitted with a non-linear equation, as shown in Figure 7, which indicates that the distributions of free and bound Cl^−^ in cement paste are significantly affected by the total Cl^−^ content. As the content of total Cl^−^ increases, the free Cl^−^ content also increases, and this increase is rapid and then slows down, while the content of bound Cl^−^ shows an “S” curve of first increasing, then decreasing, and then increasing again. When the content of total Cl^−^ is low (within 2%), the content of bound Cl^−^ in the cement paste is greater than that of free Cl^−^, which indicates a strong binding capacity for Cl^−^. As the content of total Cl^−^ ranges from 2% to 4%, the free Cl^−^ content is significantly higher than the bound Cl^−^ content. The binding capacity of Cl^−^ in cement paste is weak at this Cl^−^ content range. However, as the total Cl^−^ content increases further, the bound Cl^−^ content gradually increases, and the rate of increase in the content of free Cl^−^ slows down. This indicates an improvement in the capacity of cement paste to bind Cl^−^.

**Figure 8:**
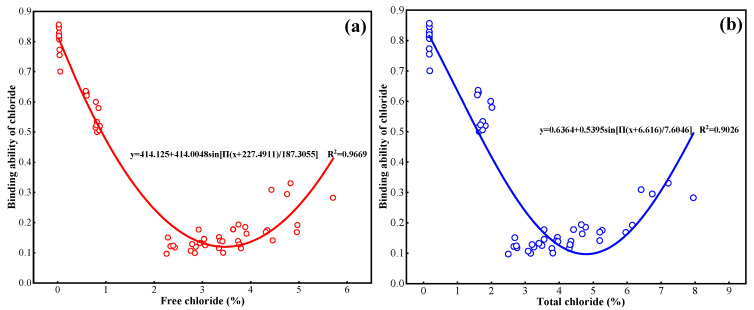
Relationship between (**a**) free Cl^−^, (**b**) total Cl^−^ and binding capacity of Cl^−^ in cement paste.

The binding capacity of Cl^−^ in cement paste is expressed by calculating the ratio of bound Cl^−^ to total Cl^−^. The relationship between the chloride binding capacity and the content of free Cl^−^, total Cl^−^ is fitted using a non-linear equation, as illustrated in Figure 8a,b. The relationship curves between the content of free Cl^−^, total Cl^−^ and the chloride binding capacity exhibit a consistent pattern of change. Specifically, as the content of free Cl^−^ and total Cl^−^ increases, the binding capacity of Cl^−^ in the cement paste initially decreases and then increases. Overall, the rise in both free and total Cl^−^ levels has an adverse impact on the ability of the cement paste to bind Cl^−^. However, this negative effect becomes smaller as the content of Cl^−^ continues to rise.

### 3.6. DTG Analysis

**Figure 9:**
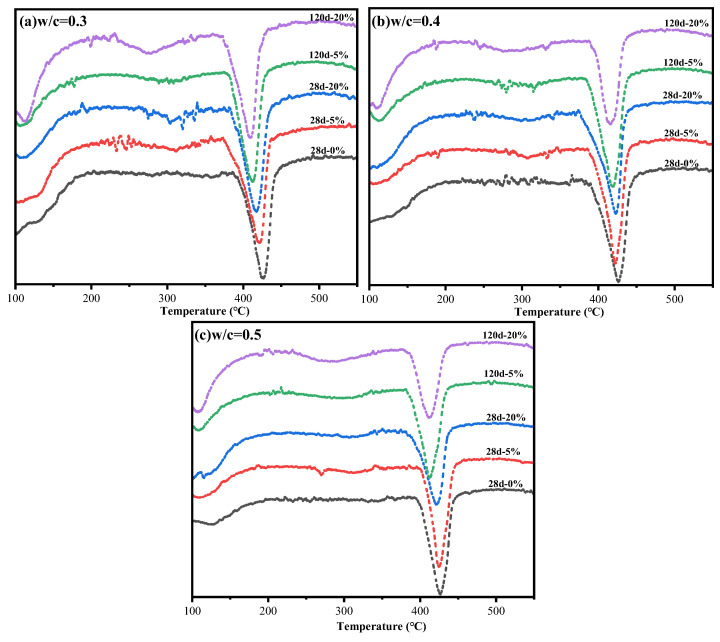
DTG results for cement paste at (**a**) W/C = 0.3, (**b**) W/C = 0.4, (**c**) W/C = 0.5.

DTG analyses are conducted on cement paste samples that had been cured for 28 days and 120 days. The results, displayed in Figure 9, indicate that the decomposition peaks of Fs were observed at approximately 300 °C, while calcium hydroxide (CH) exhibited peaks at around 430 °C [38]. These findings confirm that the characteristic peaks observed in XRD analyses at approximately 11° represent Fs. At a curing age of 120 days and a premixed Cl^−^ concentration of 20%, the decomposition of Fs causes a more significant change in the curve. This suggests that the content of Fs as a proportion of hydration products increases as the curing age and content of Cl^−^ increases. However, additional research is needed to investigate the relationship between Cl^−^ bound by Fs and the total bound chloride.

The peak area of the DTG curve was calculated to determine the mass loss of the corresponding hydration product. The dehydration peak of Fs is caused by the release of the main layer of water, and Fs dehydration’s mass loss can be determined by subtracting the mass loss of paste with NaCl from pure paste in the TG curve in the range 210–360 °C [10,38]. According to the relevant research of Zhenguo Shi [10] and Zirui Cheng [38], the molecular structure of Fs indicates that the main layer contains six water molecules. Formulae (4) and (5) were used to determine the content of Fs and chemically bound Cl^−^, respectively, since the strength of the Fs dehydration peak was associated with the release of six water molecules in the main layer:

(4) $$w_{Fs}=\frac{\Delta m_{Fs}\times \frac{M_{Fs}}{6M_{H_{2}O}}}{M}\times 100\%$$

(5) $$C=w_{Fs}\times \frac{2M_{Cl}}{M_{Fs}}$$

where $\Delta m_{Fs}$ represents the mass loss of Fs corresponding to the TG curve, $M_{Fs}$, $M_{H_{2}O}$ and $M_{Cl}$ are the molar masses of Fs, H_2_O and Cl^−^, respectively, and M represents the mass of the sample used for the TG test.

**Figure 10:**
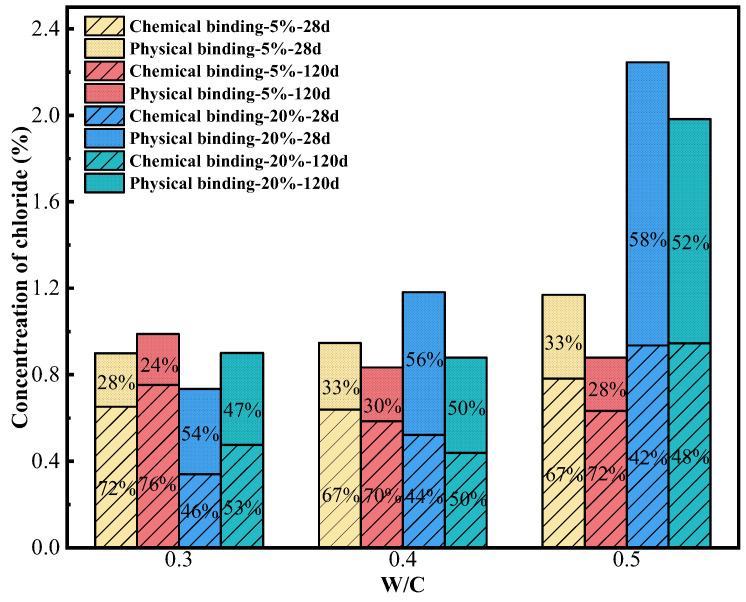
The content of physical adsorption and chemical binding of Cl^−^ in cement pastes.

**Figure 11:**
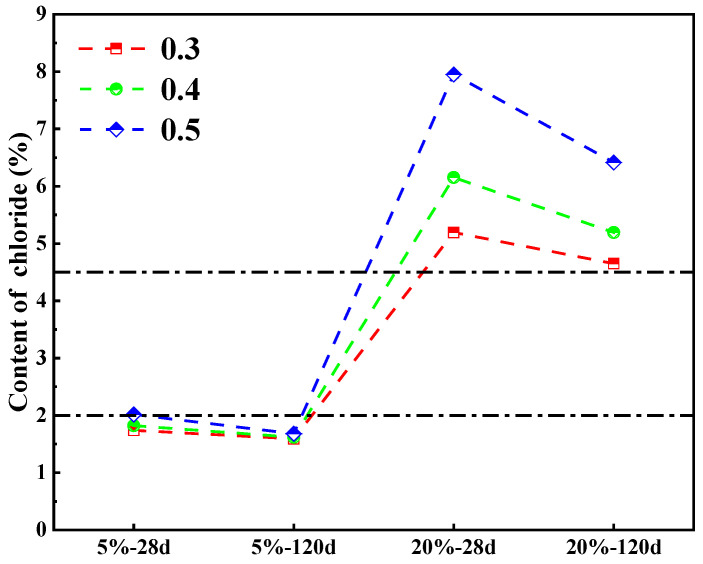
The content of total Cl^−^ in cement pastes.

The content distribution of Cl^−^, both chemically bound and physically adsorbed in the cement paste, was calculated out by the total bound Cl^−^ content. This content of chemical binding and physical adsorption of Cl^−^ is illustrated in Figure 10. At the same time, in combination with the total Cl^−^ content depicted in Figure 11, it can be obtained that when premixed with a 5% concentration of Cl^−^, the total Cl^−^ content in the cement paste remains below 2% across all W/C, with the ratio of chemically bound Cl^−^ to total bound Cl^−^ oscillating around 70%. And the proportion of chemically bound Cl^−^ increases as the hydration reaction progresses with time. When the cement paste is premixed with a 20% concentration of Cl^−^, the content of total Cl^−^ exceeds 4.5%, and the proportion of the chemically bound Cl^−^ in the cement paste to the total bound Cl^−^ decreases significantly, to around 40–50%, while the content of physically adsorbed Cl^−^ increases significantly. This suggests that the increase in concentration of Cl^−^ inhibits the formation of Fs. The capacity of Cl^−^ to bound chemically decreases as the content of total Cl^−^ increases. In contrast, the increase in the content of Cl^−^ promotes the adsorption of Cl^−^ by C-S-H and increases the amount of Cl^−^ adsorbed per unit of C-S-H [39]. In addition, when the content of total Cl^−^ in the cement paste exceeds 4.5%, the content of chemically bound Cl^−^ is elevated as the content of total Cl^−^ continues to increase. This indicates that a continuous rise in the content of Cl^−^ does not lead to a persistent decline in the content of Fs, and that the proportion of Cl^−^ bound by Fs to total bound Cl^−^ initially decreases and then stabilizes as the content of Cl^−^ increases.

In summary, Friedel’s salt, as one of the hydration products of cement-based materials (such as reinforced concrete) in chloride environments, is the main form of chemically bound Cl^−^. When the Cl^−^ concentration in the environment is high, such as in salt lakes or salt ponds, Cl^−^ ions in the environment enter concrete through pore solutions, replacing the original anions (such as SO_4_^2−^) in the AFm phase structural layers, forming new and more stable compounds, i.e., Friedel’s salts (AFm-Cl phase). This process directly changes the form and distribution of Cl^−^ in the system: on the one hand, it reduces the concentration of free Cl^−^. The Cl^−^ ions that can move freely in the solution are “captured” or “fixed” into the crystal structure of Friedel’s salt through the above reaction. This directly reduces the concentration of free Cl^−^ in the pore solution. Free Cl^−^ ions are the main cause of steel corrosion, so this process is crucial for delaying steel corrosion. On the other hand, when the bound Cl^−^ concentration increases, Cl^−^ ions fixed to the Friedel’s salt crystal structure lose their ability to move freely and engage in electrochemical activity, and temporarily do not participate in corrosion reactions. Therefore, enhancing the ability of cement-based materials to bind Cl^−^ is of great research significance for delaying steel corrosion and prolonging the durability of reinforced concrete structures.

## 4. Discussion

In order to explore the erosion mechanism of Cl^−^ on steel-reinforced concrete, many scholars have conducted extensive research using Cl^−^ concentration as a variable, believing that the capacity of cement-based materials to bind Cl^−^ enhances with increasing content of Cl^−^. However, the majority of these investigations are predicated on external infiltration tests, as the Cl^−^ diffuses from the surface of concrete to the interior, and the concentration does not exceed 5%, making it difficult to represent the limit of the cement’s ability to bind Cl^−^. This study investigates the binding behavior of cement with Cl^−^ by premixing a substantial quantity of Cl^−^ ions at a minimum concentration of 5% to facilitate their involvement in the cement’s hydration reaction. With premixed Cl^−^, we noticed that the Cl^−^ within the cement pastes precipitates outward during the curing process, contrasting sharply with the conditions resulting from external infiltration. This precipitation phenomenon becomes more pronounced with a decreasing W/C and an extended curing duration. When the concentration of premixed Cl^−^ attains 10% or more, the binding capacity of Cl^−^ is markedly influenced, particularly with regard to the impact on the chemical binding of Cl^−^. Interestingly, as the concentration of premixed Cl^−^ further increases, the ability of the cement to bind Cl^−^ shows an upward trend, which is potentially attributable to the augmented binding sites resulting from matrix cracking. However, at this stage, the cement paste matrix is significantly compromised. In regions with limited fresh water, a Cl^−^ concentration of less than 5% in mixing water is acceptable for the cement matrix, and the binding effect of Cl^−^ has a certain strengthening effect on the matrix. However, with the increase in Cl^−^ concentration, the binding ability of the hydration products of cement-based materials reaches the upper limit, and more free Cl^−^ ions exist in the pores of reinforced concrete structures. Corrosion of steel bars is still inevitable. Therefore, the improvement of the binding ability essentially increases the critical Cl^−^ concentration threshold and delays the occurrence of corrosion. These research results can provide a theoretical basis for concrete design in areas with high concentrations of salt, such as salt lakes.

## 5. Conclusions

(1) Cl^−^ can contribute to the hydration reaction of cement, which produces the AFm phase. When a 5% concentration of Cl^−^ is premixed into the cement paste, it can improve its compressive strength. When the W/C is 0.3, the effect is most pronounced. When the concentration of premixed Cl^−^ exceeds 5%, the strength of the cement paste is negatively impacted. This leads to the formation of a significant amount of NaCl crystals and excessive Fs, which can damage the cement paste’s structure. When the W/C is 0.5, the adverse effects of Cl^−^ are minimized.
(2) The content of total Cl^−^ greatly influences the binding ability of Cl^−^, and the W/C and curing age indirectly influence the binding capacity of Cl^−^ by impacting the total Cl^−^ content. When the concentration of premixed Cl^−^ is 5%, the cement paste contains approximately 2% total Cl^−^ and exhibits strong Cl^−^ binding capacity. However, when the content of total Cl^−^ in the cement paste surpasses 2%, the binding capacity for Cl^−^ decreases significantly. The binding capacity of Cl^−^ in cement paste continues to decrease as the content of total Cl^−^ increases in the range of 2% to 4%. Interestingly, when the content of total Cl^−^ in the cement paste exceeds 4%, the binding capacity for Cl^−^ increases as the content of total Cl^−^ increases. Furthermore, the structural integrity of the cement paste may also play a role in determining its ability to bind with Cl^−^.
(3) As the amount of total Cl^−^ in cement paste increases, the amount of free Cl^−^ in cement paste also increases, with a rapid initial increase followed by a slower increase, while the content of bound Cl^−^ initially decreases and subsequently increases as the content of total Cl^−^ increases. On the whole, a rise in the content of Cl^−^ has a detrimental effect on the binding of Cl^−^.
(4) The rise in content of Cl^−^ promotes the adsorption of Cl^−^ by C-S-H, whereas the proportion of Cl^−^ associated with Fs to the total bound Cl^−^ first declines and eventually tends to stabilize as the content of Cl^−^ increases. When the content of total Cl^−^ in the cement paste reaches 2%, chemically bound Cl^−^ constitutes about 70% of the total bound Cl^−^. However, when the content of total Cl^−^ exceeds 4.5%, the proportion of chemically bound Cl^−^ diminishes to 40–50%, with physically adsorbed Cl^−^ becoming predominant.
(5) When the premixed Cl^−^ concentration is 5%, the cement paste has better mechanical properties and increased binding abilities of Cl^−^. However, a premixed concentration of 5% is only the “safety limit” for cement paste. For reinforced concrete structures, when the concentration of free Cl^−^ is higher than the normative thresholds, it should not be underestimated.
(6) The Cl^−^ binding ability of the hydration products of cement-based materials was explored through premixed high concentration Cl^−^ tests. The research results can provide reference for the performance and durability design of cement-based materials in areas such as salt ponds and high salinity lakes.

## Figures and Tables

**Table 1:** Chemical composition of OPC (%).

CaO	SiO_2_	Al_2_O_3_	Fe_2_O_3_	MgO	Na_2_O and K_2_O	SO_3_	Other
62.40	19.94	5.26	2.98	3.78	1.13	3.00	1.51

**Table 2:** Performance parameters of OPC.

Water Requirement of Normal Consistency (%)	Density (g/cm^3^)	Setting Time (min)		Flexural Strength (MPa)		Compressive Strength (MPa)	
		Initial Setting	Final Setting	3d	28d	3d	28d
27.6	3.1	178	228	3.5	6.7	21.2	48.6

**Table 3:** Quantities of materials.

W/C	Cement (kg/m^3^)	Water (kg/m^3^)
0.3	1715.38	514.62
0.4	1478.57	591.43
0.5	1300	650

## Data Availability

The original contributions presented in this study are included in the article. Further inquiries can be directed to the corresponding author.
